# The Evolving Experience and Outcomes of Pediatric Kidney Transplant in Abu Dhabi, UAE (2010–2024)

**DOI:** 10.1155/ijne/5188212

**Published:** 2025-12-29

**Authors:** Ela Beyyumi, Watfa Al Dhaheri, Niaz Ahmad, Muhammad Badar Zaman, Eihab Al Khasawneh

**Affiliations:** ^1^ Department of Pediatric Nephrology, Sheikh Khalifa Medical City, Abu Dhabi, UAE, skmc.ae; ^2^ Department of Surgery, Division of Transplantation, Sheikh Khalifa Medical City, Abu Dhabi, UAE, skmc.ae

**Keywords:** alloantibodies, donor-specific antibodies (DSA), graft rejection, HLA, immunosuppressants, kidney transplant, pediatric

## Abstract

**Background:**

Kidney transplant is acknowledged as the treatment of choice for end‐stage renal disease (ESRD). This study reports on the outcome of pediatric renal transplant at a tertiary hospital in Abu Dhabi.

**Methods:**

It is a retrospective study of all pediatric renal transplants performed at a single designated pediatric center between February 2010 and February 2024, including children aged 1–16 years.

**Results:**

Sixty‐nine (44% female) pediatric renal transplants were performed, 36 from living‐related donors and 33 from deceased donors. The mean age at transplant and last follow‐up were 9.8 ± 3.6 years and 13.8 ± 4.7 years, respectively. ESRD etiologies included congenital anomalies of the kidney and urinary tract (39%), nephronophthisis (19%), glomerulonephritis (13%), and other causes (29%). Thirteen (19%) children underwent a preemptive transplant, whereas 56 (81%) were on dialysis at transplant. Thirty‐one (45%) children had graft rejection: 16 (23%) in the first year, 9 (13%) in years 2–5, and 6 (9%) thereafter. Donor‐specific antibodies (DSAs) were detected in 19 (28%) children; 17 (25%) of those had graft rejection with anti‐DR or anti‐DQ alloantibodies. Fourteen children had DSA‐negative graft rejection. Of those, eight had cell‐mediated rejection, and six had mixed rejection. Predictors of rejection were positive DSA (*p* = < 0.0001) and two DR mismatches (*p* = 0.029); three graft losses occurred. The prevalence of EBV, CMV, and BKV infection in the first year was 43%, 39%, and 33%, respectively, falling to 38%, 18%, and 12% in the subsequent years. Thirty‐four (49%) children had at least one episode of culture‐positive urinary tract infection. The 1‐year and 5‐year patient survival rates were 100% and 96.6%, and the corresponding graft survival rates were 98.1% and 89.7%, respectively.

**Conclusion:**

The outcome of pediatric kidney transplants in Abu Dhabi over 14 years shows patient and graft survival comparable to published data. Acute graft rejection remains a major challenge with the presence of DSA and biallelic HLA‐DR mismatch as independent predictors for rejection. Optimizing donor selection, immunosuppression, and closer surveillance are vital.

## 1. Introduction

The long‐term survival of pediatric kidney grafts varies markedly among different studies [1]. Recent reports however show consistent improvements in graft survival, mainly attributed to better donor selection, more advanced surgical skills, innovative antirejection treatment, implementing regular surveillance, and adherence to essential supportive care measures [2, 23].

The pediatric renal transplant service was established in 2010 at Sheikh Khalifa Medical City (SKMC), a tertiary care pediatric hospital in Abu Dhabi, UAE. The outcome of pediatric renal grafts was first reported in 2019 [3]. One‐ and 5‐year patient survival of 100% and 96.7%, and graft survival of 96.7% and 83.3% were reported. Rejection episodes were 16.7% [3]. These results have been consistent with other regional centers [4, 5, 24]. However, the deceased donor kidney transplant program was established in the UAE in 2017. The population of the UAE in general and the donor population comprise multiple ethnicities. While this presents opportunities for transplant due to the acceptance of organ donation by many ethnic groups, the optimal human leukocyte antigen (HLA) matching poses a challenge for the same reason [25]. Since then, the transplant service has aimed for close HLA matching between the donor and the recipient. Additionally, surveillance for preformed anti‐HLA antibodies and donor‐specific antibodies (DSAs) is conducted pre‐ and posttransplant. Routine graft biopsies are performed for all children with impaired function who are also positive for DSA. Therefore, we expect the prevalence of rejection to be higher than in the earlier part of our experience [3]. Thus, the study aims to update and reevaluate the outcome of pediatric kidney transplants at SKMC, reporting long‐term patient and graft survival, the incidence of allograft rejection, and exploring variables amenable to improvements.

## 2. Methods

This retrospective study was conducted at (SKMC), Abu Dhabi, United Arab Emirates. The study was approved by the SKMC Research Ethics Committee (857). Informed consent to participate in this retrospective chart review was exempted. Transplant‐related data were prospectively maintained in the hospital electronic record. The follow‐up data were retrospectively collected from patients’ medical records.

Since 2010, 580 adult and 78 pediatric renal transplants have been performed at SKMC.

The objective of the study was to describe the clinical course of pediatric kidney transplantation, with emphasis on the incidence of acute graft rejection and factors affecting graft rejection and subsequent outcome. All children aged 1–16 years who underwent kidney transplantation at our center between February 2010 and February 2024 were included. They were followed up for the duration of the study. All the transplants were ABO‐compatible. High‐resolution HLA‐A, HLA‐B, HLA‐C, HLA‐DR, and HLA‐DQ were determined for recipients and donors. Following the transplant, recipients had regular surveillance (monthly for the first year, every 3 months for the second year, then biannually) for DSAs against mismatched alleles, using Luminex‐detected HLA antibodies. Graft biopsy was performed for positive DSA and/or impaired graft function. The latest Banff criteria at the time of biopsy were used to determine rejection classification and severity [6].

For induction [7, 8], typically antithymocyte globulin (rabbit), ATG, at 1.5 mg/kg/day for 3–5 days (first dose on the day of transplant), was given for recipients with high immunologic risk, characterized by deceased donor, high number of HLA mismatches, and the presence of preformed DSA. ATG for 5 days was given for children with a lymphocyte count > 0.1 × 10^9^/L or CD3 count > 100/μL on Day 4 [9]. Twenty‐five children received ATG; their cumulative dosing was 5.5 ± 1.1 mg/kg (median, 5.5; range 3–7.5). For these children, an alternative to ATG, alemtuzumab (a monoclonal antibody that binds CD52), a single intraoperative dose of 0.6 mg/kg (maximum, 12 mg), was considered for induction. For other children deemed to have a low immunological risk, particularly those receiving a graft from a living donor, induction with basiliximab (a chimeric anti‐interleukin‐2 receptor monoclonal antibody), 10 mg for children weighing less than 35 kg and 20 mg for those weighing more than 35 kg, was given. The first dose was administered intraoperatively and the second dose on Day 4. In addition, methylprednisolone (10 mg/kg) was given to all children regardless of their risk status.

Maintenance regimen typically consisted of tacrolimus, mycophenolate mofetil (MMF), and prednisolone. Tacrolimus (calcineurin inhibitor) with a target trough level of 10–15 ng/mL in the first month, 8–12 ng/mL in months 2–3, 6–8 ng/mL from months 4–6, and 4–6 ng/mL thereafter. MMF started at 600 mg/m^2^/dose twice daily. Intravenous methylprednisolone at 5 mg/kg was given on postoperative Day 1 and reduced to 1 mg/kg by Day 4, then switched to oral prednisolone and gradually tapered in the outpatient setting to 0.07–0.1 mg/kg by 3–4 months posttransplant, provided serum creatinine remained stable.

Biopsy‐proven rejection episodes were treated as per hospital protocol based on the type of rejection. Briefly, for T cell–mediated rejection, Banff IA, augmentation of maintenance immunotherapy and pulse methylprednisolone 5–10 mg/kg for 3–5 days were used first line. For refractory cases and for Banff IB, II, and III rejection, ATG was administered at either 3 mg/kg per day for 3 days or 1.5 mg/kg per day for 5–7 days (total cumulative dose 7.5‐9 mg/kg). Alemtuzumab was used occasionally as an alternative. When ATG or alemtuzumab was used, valganciclovir and co‐trimoxazole prophylaxis were administered. Additionally, routine viral monitoring for BK virus, cytomegalovirus (CMV), and Epstein–Barr virus (EBV) was escalated to monthly for the first 6 months after treatment of rejection, then every 3 months thereafter. Antibody‐mediated rejection was treated based on the DSA level and biopsy findings. The regimen consisted of pulse methylprednisolone 10 mg/kg for 3 days, high‐dose IVIG at 2 g/kg over 2 days, and a single dose of rituximab (375 mg/m^2^). For high DSA titers (>5000) with a >50% increase in creatinine, plasma exchange (1× volume exchange with albumin replacement) was performed for 5–7 sessions, along with bortezomib at 1.3 mg/m^2^ on Days 1, 4, 8, and 11.

The outcome was based on graft and patient survival. A graft loss was considered in patients who returned to dialysis posttransplant. An impaired graft function was considered in children with a decreased estimated glomerular filtration rate (eGFR) < 60 mL/min/1.73 m^2^ using the Schwartz formula. Delayed graft function (DGF) is defined as the requirement of dialysis within 1 week posttransplant.

The statistical analysis was performed on SPSS (Version 21). Categorical variables were compared using chi‐square or Fisher’s exact test. Comparisons of mean eGFR at 1 and 5 years among induction groups were performed using one‐way ANOVA. Multiple logistic regression of rejection versus predictors was performed using backward selection (likelihood ratio). The Mann–Whitney test was used to compare eGFR medians between children who had graft rejection and those who did not. *P* < 0.05 was considered significant.

## 3. Results

Sixty‐nine children underwent renal transplants since 2010, and all recipients were of their first transplant. Their characteristics are presented in Table 1. Thirty‐six children had their grafts from a living‐related donor and 33 from a deceased donor. Thirteen (19%) children underwent a preemptive transplant, whereas 56 (81%) children were on dialysis at the time of transplant. The duration of dialysis was 21.2 ± 17.0 (mean ± SD) months in children receiving transplant from living donors and 62.6 ± 35.8 (mean ± SD) months for those receiving deceased donor grafts. For preemptive transplantation, the average time from the diagnosis of Stage 5 kidney disease to transplantation was 14.9 ± 16.2 (mean ± SD) months.

**Table 1:** The demographic characteristics of the recipients of the first kidney transplant during the study period from Feb 2010 to Feb 2024 (*n* = 69).

	Renal graft rejection	
	Yes (*n* = 31)	No (*n* = 38)
Age (y)		
Mean ± SD	14.8 ± 4.2	13.0 ± 5.6
Median	14	13
Range	7–23	4–25
Age at transplantation (y)		
Mean ± SD	10.0 ± 3.4	9.7 ± 3.8
Median	11	9.0
Range	4.0 to 15.0	4.0 to 16.0
Gender		
Females	13 (19%)	17 (25%)
Males	18 (26%)	21 (30%)
Weight at transplantation (kg)		
Mean ± SD	28.2 ± 11.3	28.8 ± 14.0
Median	25	24
Range	15–60	10–70
Primary renal disease		
CAKUT	14 (20%)	13 (19%)
Nephronophthisis	4 (6%)	9 (13%)
Glomerulonephritis	4 (6%)	5 (7%)
SRNS	0 (0%)	3 (4%)
CNS	5 (7%)	4 (6%)
ARPKD	1 (1%)	2 (3%)
FHHNC, cystinosis, cortical necrosis	3 (4%)	2 (3%)
Mode of dialysis pretransplant		
Hemodialysis	15 (22%)	14 (20%)
Peritoneal dialysis	11 (16%)	16 (23%)
Preemptive	5 (7%)	8 (12%)
Major blood group		
A	6 (9%)	11 (16%)
B	6 (9%)	16 (23%)
AB	2 (3%)	3 (4%)
O	17 (25%)	8 (12%)
HLA (A, B, C, DR, DQ)		
Full match	2 (3%)	4 (6%)
Mismatch	28 (41%)	34 (50%)
Donor		
Living	14 (20%)	22 (32%)
Deceased	17 (25%)	16 (23%)
Induction		
Antithymoglobulin	10 (14%)	15 (22%)
Alemtuzumab	9 (13%)	9 (13%)
Basiliximab	10 (14%)	13 (19%)
Not documented	2 (3%)	1 (1%)
Donor‐specific HLA antibody (DSA)		
Positive^(1)^	17 (25%)	2 (3%)
Negative	14 (20%)	36 (52%)
Delayed graft function (DGF)		
Yes	1 (1%)	2 (3%)
No	30 (43%)	36 (52%)
Number of rejections		
One	15 (22%)	—
Two	9 (13%)	
Three or more	7 (10%)	
Time to first rejection (overall, 45% rejection)		
< 6 months	11 (16%)	—
6 months to 1 year	5 (7%)	
1–3 years	7 (10%)	
3–5 years	2 (3%)	
> 5 years	6 (9%)	
Mechanism of first rejection		
Antibody‐mediated	3 (4%)	—
Cell‐mediated	11 (16%)	
Both	17 (25%)	
EBV risk stratification, donor (D) versus recipient (R) serology		
Low (D‐/R‐)	0 (0%)	1 (1%)
Medium (D+/R+) or (D‐/R+)	21 (30%)	29 (42%)
High (D+/R‐)	10 (14%)	8 (12%)
CMV risk stratification, donor (D) versus recipient (R) serology		
Low (D‐/R‐)	1 (1%)	0 (0%)
Medium (D+/R+) or (D‐/R+)	27 (39%)	30 (43%)
High (D+/R‐)	3 (4%)	8 (12%)
Infections during the first year posttransplant		
CMV	12 (18%)	14 (21%)
EBV	12 (18%)	17 (25%)
BK	16 (24%)	6 (9%)
Infections after the first year posttransplant		
CMV	10 (15%)	2 (3%)
EBV	13 (20%)	11 (18%)
BK	8 (12%)	0 (0%)
Urinary tract infections since transplant	18 (26%)^∗^	16 (23%)

*Note:* Recipients are divided into rejection and no rejection groups.

Abbreviations: ARPKD, autosomal recessive polycystic kidney disease; BK, polyomavirus; CAKUT, congenital anomalies of the kidney and urinary tract; CMV, cytomegalovirus; CNS, congenital nephrotic syndrome; EBV, Epstein–Barr virus; FHHNC, familial hypomagnesemia with hypercalciuria and nephrocalcinosis; SRNS, steroid‐resistant nephrotic syndrome.

^∗^Twelve children had UTIs at least 6 months before the rejection episodes.

^(1)^ *P*‐value between the groups is < 0.0001.

The etiologies of end‐stage renal disease (ESRD) in Table 1 included congenital anomalies of the kidney and urinary tract, nephronophthisis, glomerulonephritis, steroid‐resistant nephrotic syndrome, congenital nephrotic syndrome, autosomal recessive polycystic kidney disease, familial hypomagnesemia with hypercalciuria and nephrocalcinosis, cystinosis, and cortical necrosis. A total of 29 children had genetic testing, typically multiplex ligation‐dependent probe amplification (MLPA) followed by whole exome sequencing or a specific renal panel. Results of 17 children were available and are presented in Table 2. Diagnoses of the remaining children included congenital nephrotic syndrome (MIM#256300), Pierson syndrome (MIM#609049), Bardet–Biedl syndrome 20 (MIM#619471), autosomal recessive polycystic kidney disease (MIM#263200), CLAUDIN 19 (MIM#248190, hypomagnesemia 5, renal, with ocular involvement), Joubert syndrome, and Prune belly syndrome (MIM#100100).

**Table 2:** Fate of the pediatric renal transplants by the identified genetic variants (*n* = 17).

Patients	Genetic variants	In silico Prediction^1^	Renal graft rejection
1	NM_025114.3(*CEP290*):c.4792_4795delAAAT, p.K1598Sfs∗8 (#MIM 610142), Joubert syndrome 5 (MIM#610188, AR), homozygous.	Pathogenic	Yes
2	Two‐copy number loss of exons 12–15 of *CEP290* (MIM# 610142), Joubert syndrome 5 (MIM# 610188, AR), homozygous.	Pathogenic	Yes
3	(a) (*CLDN19*):c.241C>T, p.R81W (MIM#610036), hypomagnesemia 5, renal, with ocular involvement (MIM#248190, AR), homozygous. (b) *COL1A1*:c.1249C>G, p. P417A (MIM#120150), osteogenesis imperfecta, type I (MIM#166200, AD), heterozygous.	Pathogenic	Yes
4	(*CTNS*):c.422C>T, p.S141F (MIM# 606272), cystinosis, nephropathic (MIM#219800, AR), homozygous	Pathogenic	Yes
5	(a) NM_003647.2(*DGKE*):c.953A>G, p.N318S (MIM#601440), nephrotic syndrome, type 7 (MIM#615008, AR), homozygous. (b) NM_212482.2(*FN1*):c.5587C>T, p.P1863S (MIM#135600), glomerulopathy with fibronectin deposits 2 (MIM#601894, AD), heterozygous.	Damaging	No
6	NM_004646.3 (*NPHS1*) c.1758–8T>G (MIM#602716), nephrotic syndrome, Type 1 (MIM#256300, AR), homozygous.	Disease causing	No
7	On one chromosome, a large deletion encompassing the entire *CFHR1* and *CFHR3* has been identified, and on the other chromosome, a large deletion encompassing the entire *CFHR1* has been identified: One copy of *CFHR3* is present, and both copies of *CFHR1* are deleted. These findings were confirmed by MLPA. Therefore, the increased genetic susceptibility to atypical hemolytic uremic syndrome is confirmed.	Disease causing	No
8	(a) MLPA: Heterozygous deletion of *CFHR1* (MIM# 34371) and *CFHR3* (MIM#605336), hemolytic uremic syndrome, atypical, susceptibility to (MIM#: 235,400, AD AR), heterozygous. (b) NM_001710.5(*CFB*):c.1697A>C, p.E566A (MIM#138470), hemolytic uremic syndrome, atypical, susceptibility to, 4 (MIM#612924, AD), heterozygous. (c) NM_139025.3 (*ADAMTS13*):c.2915G>A, p.R972Q (MIM#604134), thrombotic thrombocytopenic purpura, hereditary (MIM#274150, AR), heterozygous	Pathogenic	No
9	(*NPHS2*):c.779T>A, p.V260E (MIM#604766), nephrotic syndrome type 2 (MIM# 600995, AR), homozygous	Pathogenic	No
10	(a) NM_014425(*INVS*):c.2866G>A, p.E956K, rs114749415 (MIM# 243305), nephronophthisis 2, infantile (MIM#602088, AR), homozygous. (b) NM_002942 (*ROBO2*):c.2390G>A, p.R797Q (MIM# 602431), vesicoureteral reflux 2 (MIM#610878, AD), heterozygous. (c) NM_033087(*ALG2*):c.29_31delACT, p.D10_S11delinsA, congenital disorder of AR glycosylation, Type Ii (CDG1I) [MIM:607906]; myasthenic syndrome, congenital, 14, with tubular aggregates [MIM:616228]. (d) NM_015559(*SETBP1:*c.1352T>C, p.I451T, novel variant, mental retardation, autosomal AD dominant 29 [MIM:616078]; Schinzel–Giedion midface retraction syndrome [MIM:269150] Chr18: p.I451T.	Disease causing	No
11	NM_003990.4(*PAX2*):c.360del, p.E121Rfs∗38 (MIM# 167409), papillorenal syndrome (MIM#120330, AD), heterozygous	Pathogenic	No
12	Steroid‐resistant nephrotic syndrome (MIM#600995) with homozygous variant in *NPHS2* (podocin), MIM#604766; report not available.	_	No
13	(a) NM_014714(*IFT140*): exon 31 deletion (homozygous), short‐rib thoracic dysplasia 9 with or without polydactyly (MIM#266920, AR). (b) NM_006269(*RP1*)*:*c.4735T>G, L1579V, retinitis pigmentosa 1 (MIM#180100, AD), homozygous. (c) NM_020937(*FANCM*):c.2859A>C, p.K953N (MIM#609644), Fanconi anemia complementation group M (MIM#614087, AR), homozygous.	Damaging	No
14	NM_000091.3(*COL4A3*):c.2636C>T, p.P879L (MIM#120070), Alport syndrome 3A, (MIM# 104200 AD), Alport syndrome 3B(MIM#620536 AR), heterozygous	Disease causing	No
15	Homozygous deletion of the chromosomal region chr2:110849180–110970348 encompassing the entire *NPHP1* (MIM#607100), nephronophthisis 1, juvenile (MIM#256100, AR).	Pathogenic	No
16	*COL4A3* (MIM#120070) homozygous variant; report not available.	_	No
17	Homozygous variant in *NPHS1* (MIM#602716), nephrotic syndrome, type 1 (MIM#256300, AR); report not available.	_	No

*Note:* Twenty‐four pathologic variants were detected in 17 children, and 14 (58%) were homozygous, thus amenable to prevention through premarital screening and counseling. Seven variants were single‐nucleotide polymorphisms, 3 large deletions, and 2 frameshifts. Twelve additional patients had genetic tests, but their reports are not available or the results are negative; their diagnoses included the following: congenital nephrotic syndrome (MIM#256300), Pierson syndrome (MIM#609049), Bardet–Biedl syndrome 20 (MIM#619471), ARPKD (MIM#263200), CLAUDIN 19 (MIM#248190, hypomagnesemia 5, renal, with ocular involvement), Joubert syndrome, and Prune belly syndrome (MIM#100100). Gene symbols are in italics, and novel variants are shown in bold.

Abbreviations: AR, autosomal recessive; AD, autosomal dominant; MLPA, multiplex ligation‐dependent probe amplification.

^1^The most severe prediction is listed.

Twenty‐four pathologic variants were detected in 17 children, and 14 (58%) were homozygous, thus amenable to prevention through premarital screening and counseling. Five (21%) of these children had multiple variants, ranging from 2 to 4. Of the detected variants, there were 15 (63%) single‐nucleotide polymorphisms (SNPs), 5 (21%) large deletions, 2 (8%) frame shifts, 1 (4%) copy number variant (CNV), and 1 (4%) intronic (Table 2).

The majority of children were of low immunological risk, with a negative panel‐reactive antibody (PRA) for Class 1 in 72.5%; the remaining had positive PRA ranging from 1.0% to 12.0%. Similarly, 71% had negative PRA for Class 2, while the remaining had positive results from 1.0% to 22.0%. Preformed DSA was identified in two (2.9%) children prior to transplant. Mean HLA mismatch for HLA‐A, HLA‐B, and HLA‐DR was 3.35. Only 6 children had full matches of the HLA‐A, HLA‐B, HLA‐C, HLA‐DR, and HLA‐DQ; the remaining had various mismatches, including 19 (29%) with biallelic HLA‐DR mismatch as summarized in Table 3.

**Table 3:** Human leukocyte antigen disparities (HLA‐A, B, C, DR, and DQ loci) between the recipients of renal grafts and the donors, sorted by graft rejection (*n* = 65).

Mismatches		Graft rejection					
		Yes			No		
		0	1	2	0	1	2
Overall	A	6	12	9	6	23	9
	B	4	13	10	9	13	16
	C	4	9	13	9	14	11
	DR**^∗^**	4	11	12	11	20	7
	DQ	9	10	7	17	11	7

Living donor	A	5	5	0	6	15	1
	B	3	7	0	9	10	3
	C	4	5	0	7	11	1
	DR	3	7	0	10	11	1
	DQ	5	4	0	15	5	0

Deceased donor	A	1	7	9	0	8	8
	B	1	6	10	0	3	13
	C	0	4	13	2	3	10
	DR	1	4	12	1	9	6
	DQ	4	6	7	2	6	7

*Note:* 0, no mismatch; 1, one allelic mismatch; 2, biallelic mismatches.

Data were unavailable in four children. Six children had a full match (scored 0 for each of the five loci). Three children had a full mismatch (scored 2 for each of the five loci). Nineteen children had two mismatches in the DR locus.

^∗^ *p*‐value for the presence of biallelic mismatches (score 2) for the DR locus between the two groups (rejection vs. no rejection) was 0.029. In contrast, the corresponding *p*‐value for the DQ locus is 0.553.

Biallelic mismatches between the rejection and no rejection group were significant for HLA‐DR (*p*‐value 0.029) and not for HLA‐DQ (*p*‐value 0.553). The significance was also consistent for the presence of DSA in the two HLA groups, HLA‐DR biallelic mismatch being significantly associated with the presence of DSA (*p*‐value 0.007) compared with biallelic mismatch at HLA‐DQ (*p*‐value 0.092).

The mean cold ischemia time was 1 h 20 min for living donors and 6 h 17 min for deceased donor grafts. Postoperative complications included DGF in three children, bladder rupture with urine leak in one, renal artery stenosis requiring angioplasty in one, and vascular thrombosis in one child.

Twenty‐five (35%) children were induced with ATG, while 18 (26%) and 23 (33%) were induced with alemtuzumab and basiliximab, respectively. There was a total of 31 (45%) graft rejections, 14 from living and 17 from deceased with no statistically significant difference between the donor type and rejection (*p*‐value 0.3). Time to rejection was 16 (23%) in the first year, 9 (13%) in years 2–5, and 6 (9%) thereafter (Table 1). Among the first episode of rejections, 3 were antibody‐mediated, 11 were cell‐mediated, and 17 were mixed rejections. There was no statistically significant association between induction therapy and rejection (*p*‐value 0.8).

DSAs were detected in 6 (17%) of the 36 living donor grafts versus 13 (39%) of the 33 deceased donor grafts. Importantly, rejection was documented in 17 children with positive DSA. Two children who had positive DSA (DQ [1747] and DR [1387]) had negative rejection on the kidney biopsy. These two children were not treated for the positive DSA, and follow‐up titers became negative over time.

The results of 17 children who had positive DSA with graft rejection are summarized in Table 4. Median time to the first positive DSA was 14.5 months (range 1–82 months) posttransplant. Three children had positive DSA within the first 2 months, and nine children within the first 12 months. In six children, the positive DSA was noted at or after 23 months. Six children had 2–4 separate time windows for DSA detection. The positive DSA status was converted to negative in 6 (38%) of the 17 treated children. All 17 children had anti‐DR or anti‐DQ, and 15 (88%) of the 17 children had more than one alloantibody. Of the 17 children with positive DSA, a graft loss was evident in only 2 (12%), and the remaining children were rescued with treatment (Table 4).

**Table 4:** Donor‐specific HLA antibodies (DSA) and incidence of acute graft rejection in 17 recipients.

Patients	Type of donor	HLA mismatches (A, B, C, DR, DQ)	Induction therapy	Maintenance therapy	Posttransplant DSA			Biopsy results	Treatment	DSA status after treatment	Outcome since DSA detection
					First detected (mo)	Class I alloantibodies (titer/MFI)	Class II alloantibodies (titer/MFI)				
1	Deceased	1,1,2,1,0	ATG	MMF, tacrolimus, prednisolone	1	A2 (1144), Cw15 (1522)	DQ7 (6790)	Acute TCMR Banff 1A, suspicious for ABMR	Methylprednisolone, IVIG, plasma exchange, bortezomib, rituximab	Negative	Good graft function at 8 months
2	Deceased	1,1,1,2,2	ATG	MMF, tacrolimus, prednisolone	18	_	DQ5 (1444)	Suspicious for acute ABMR	Methylprednisolone, IVIG, rituximab	Positive, DQ5 (1395)	Good graft function at 6 months
3∗	Deceased	2,1,2,2,2	Alemtuzumab	MMF, tacrolimus, prednisolone	(a) 9 (b) 20	(a) ‐ (b) A2 (7560)	(a) DQ2 (7664) (b) DR11 (3982) DR52 (13,852) DR13 (5355), DQ2 (16,739), DQ6 (11,014)	(a and b) TCMR Banff 1A, suspicious for ABMR	(a) Methylprednisolone, IVIG, plasma exchange, bortezomib. (b) Methylprednisolone, IVIG, bortezomib, ATG, plasma exchange	Not done	Good graft function at 54 months, followed by adults
4	Deceased	2,2,2,2,2	ATG	MMF, tacrolimus, prednisolone	10	_	DR53 (4031), DQ8 (823)	Suspicious for acute ABMR, suspicious for acute TCMR	Methylprednisolone, IVIG, rituximab	Positive, DR53 (722)	Good graft function at 5 months
5	Deceased	2,2,2,2,0	Alemtuzumab	MMF, tacrolimus, prednisolone	11	B58 (5187), A33 (4127), B35 (1589), Cw10 (1664),	DQ2 (28,426), DP4 (5202), DR17 (3454), DR52 (3211)	Acute active ABMR, borderline suspicious for TCMR	Methylprednisolone, IVIG, plasma exchange, rituximab, bortezomib	Negative, except for DQ2 (2545)	Good graft function at 22 months
6	Deceased	1,1,2,2,1	ATG	MMF, tacrolimus, prednisolone	11	B51 (4404), A68 (1871)	DQ6 (4907), DR51 (1664), DR15 (584)	Suspicious for ABMR, borderline suspicious TCMR	Methylprednisolone, IVIG, rituximab	Negative	Good graft function at 16 months
7	Deceased	2,2,1,1,0	Alemtuzumab	MMF, tacrolimus, prednisolone	37	Cw4 (2407)	DQ8 (884)	Inadequate for diagnosis	Methylprednisolone, IVIG, rituximab	Negative	Good graft function at 17 months
8	Mother	1,1,1,1,1	Basiliximab	MMF, tacrolimus	64	Cw16 (3035)	DQ2 (2538)	Borderline suspicious for TCMR, suspicious ABMR	Methylprednisolone	Negative for Cw16; persistent DQ2 (672) repeated biopsy at 12 months showed TCR and BK nephropathy	Impaired graft function at 23 months
9	Aunt	_	Basiliximab	Tacrolimus, azathioprine	62	_	DQ6 (7808)	Suspicious ABMR (C4d > 80%), TCMR Banff 1A	Methylprednisolone, IVIG, plasma exchange, rituximab, bortezomib	DQ6 (6450) after 1 month	Good graft function at 2 months, loss to follow‐up
10	Sibling	0,1,1,1,0	Alemtuzumab	MMF, tacrolimus, prednisolone	82	_	DQ5 (1761), DR51 (993)	Suspicious for ABMR (acute, active), borderline suspicious TCMR	Methylprednisolone, IVIG, rituximab	Not done	Good graft function at 5 months
11	Deceased	0,1,2,2,2	ATG	MMF, tacrolimus, prednisolone	2	Cw5 (546)	DQ2 (889)	Suspicious ABMR (C4d 4%). Repeated biopsy after 2 months showed suspicious ABMR (C4d 20%), suspicious borderline TCMR	Methylprednisolone, IVIG, rituximab	Negative	Impaired graft function despite negative DSA; retreated with MP, IVIG, plasma exchange, bortezomib (follow‐up; good graft function at 7 months)
12	Deceased	1,1,1,2,2	Alemtuzumab	Azathioprine, tacrolimus, prednisolone	(a) 1 (b) 11	(a) A2 (2100) (b) ‐	(a) DQ6 (2990) (b) DR52 (923)	(a) Active ABMR, borderline changes suspicious of TCMR. (b) Repeated biopsy after 11 months showed suspicious ABMR and borderline suspicious for TCMR.	(a) Methylprednisolone, plasma exchange, bortezomib. (b) Methylprednisolone, IVIG, rituximab	Negative	Good graft function at 38 months
13	Deceased	2,2,2,2,1	ATG	MMF, tacrolimus, prednisolone	(a) 7 (b) 11 (c) 15	_	(a) DQA1 (b) Negative (c) DR52 (10,163), DQ6 (7112), DP1 (4823), DR11 (2182)	(a) Suspicious acute (active) ABMR, borderline suspicious TCMR. (b) Acute TCMR, Banff 1A, suspicious ABMR. (c) Acute TCMR, Banff 1B, suspicious ABMR	(a) Methylprednisolone, plasma exchange, IVIG, rituximab (b) methylprednisolone, IVIG, bortezomib (c) methylprednisolone, IVIG, ATG, bortezomib	Negative	Impaired graft function at 12 months
14	Father	0,1,1,1,1	Basiliximab	MMF, tacrolimus, prednisolone	(a) 8 (b) 28 (c) 34	(a) Cw7 (7738) (b) Cw7 (3647) (c) Cw7 (1758)	(a) DQA1 (3835) (b)‐ (c) ‐	(a) TCMR Banff 2A, suspicious for ABMR. (b) Suspicious ABMR, borderline suspicious TCMR (c) acute (active) ABMR, TCMR Banff 1A. (d) ATCMR Banff grade 1A, chronic TCMR, chronic active ABMR	(a) Methylprednisolone, IVIG, plasma exchange, ATG, bortezomib (b) methylprednisolone, IVIG, plasma exchange, ATG, rituximab (c) methylprednisolone, plasma exchange, ATG, bortezomib (d) methylprednisolone, IVIG, ATG, eculizumab	Persistent DSA DQ2 (31,657), DR17 (2467)	Graft loss
15	Deceased	1,1,2,2,2	Alemtuzumab	MMF, tacrolimus, prednisolone	29	_	DQ5 (33,949), DQ6 (20,736), DR51(7021), DR1 (1792), 15 (1606)	Acute TCMR, Banff 1A, suspicious ABMR. Repeated biopsy showed acute TCMR, Banff 1B, suspicious ABMR	(a) Methylprednisolone (b) methylprednisolone, IVIG, plasma exchange, ATG	Persistent positive DQ5 (27,373), DQ6 (13,706), DR51 (1994), DR1 (747)	Graft loss
16	Deceased	2,2,1,2,2	ATG	MMF, tacrolimus, prednisolone	(a) Negative (b) 14 (c) 19	_	(b) DP1 (2607) (c) DP1 (4493)	(a) Borderline suspicious of TCMR, BK virus nephropathy (class 2). (b) TCMR (Banff 1A), ABMR, BK virus nephropathy. (c) ABMR, TCMR	(a) Methylprednisolone, IVIG. MMF stopped, started on leflunomide. (b) IVIG. (c) Tacrolimus changed to everolimus.	Persistent DSA DP1 (1902) DQ2(749)	Good graft function at 27 months
17	Father	1,1,0,1,1	Basiliximab	MMF, tacrolimus, prednisolone	(a) 23 (b) 26 (c) 30 (d) 36	_	(a) DQ7 (16,851) (b) DQ7 (12,249) (c) DQ7 (13,102) (d) DQ7 (10,937)	(a) ABMR (acute) and borderline changes suspicious of TCMR. (b) Repeated biopsy showed acute TCMR Banff 1A and suspicious for ABMR. (c) Repeated biopsy active ABMR. (d) Repeated biopsy active ABMR.	(a) Methylprednisolone, IVIG, bortezomib. (b) Methylprednisolone. (c) Methylprednisolone, IVIG, plasma exchange, rituximab, bortezomib (d) methylprednisolone, IVIG, rituximab	Persistent DQ7 (6287)	Impaired graft function

*Note:* Graft function reported at the time of last follow‐up. ATG, antithymocyte globulin rabbit; C4d, complement component C4d;

Abbreviations: ABMR, antibody‐mediated rejection; IVIG, intravenous immunoglobulin; MFI, median fluorescence intensity; MMF, mycophenolate mofetil; TCMR, T cell–mediated rejection.

^∗^Low DSA titer prior to the transplant.

**Figure 1:**
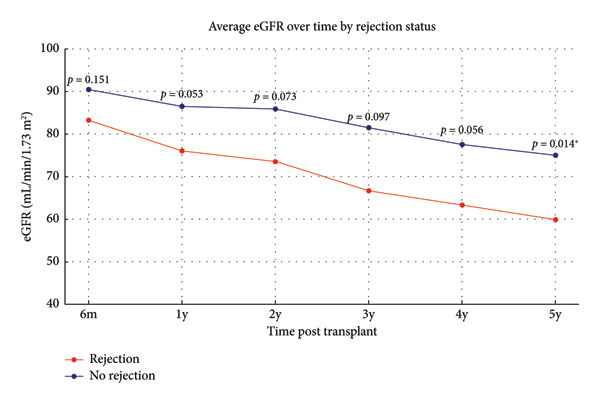
The line plot showing the mean estimated glomerular filtration rate (eGFR) in mL/min/1.73 m^2^ over a period of 5 years posttransplant comparing children who experienced rejection (red line) to those who did not (blue line). Data points represent the mean eGFR at 6 months (*n* = 68), 1 year (*n* = 55), 2 years (*n* = 46), 3 years (*n* = 39), 4 years (*n* = 30), and 5 years (*n* = 28). The corresponding *p*‐values between the groups at each time point were calculated using the Mann–Whitney test. Significant *p*‐value (< 0.05) is marked with an asterisk (^∗^).

Fourteen children had DSA‐negative graft rejection (Table 5). Median time to the first rejection postgraft transplant was 11.5 months (range 1–86 months). Four children had rejection within the first 2 months, and seven children within the first 12 months. In five children, the rejection was noted after 24 months. Six children had multiple rejections, ranging from two to five episodes. Seven children had good graft function, five had impaired graft function, one had graft loss, and one passed secondary to sepsis. Of the 14 children, 8 had cell‐mediated rejection and 6 had mixed rejection, which was confirmed by cellular rejection features and antibody‐mediated involvement, evident by C4d positivity in peritubular capillaries (Table 5). Multiple logistic regression analysis using backward selection (likelihood ratio) demonstrated a significant association between rejection and positive DSA (*p* < 0.0001). Over 5 years postrenal transplant, children with and without graft rejection exhibited a downward trend in eGFR with a more pronounced decline observed in the rejection group, at 5 years posttransplant (*p*‐value 0.014) (Figure 1).

**Table 5:** Recipients with graft rejection and negative DSA (donor‐specific HLA antibodies), *n* = 14.

Patients	Type of donor	HLA mismatches (A, B, C, DR, DQ)	Induction therapy	Maintenance therapy	Rejection detected (mo postgraft transplant)	Biopsy result	Treatment	Outcome since last rejection
1	Deceased	2,2,2,0,0	ATG	MMF, tacrolimus, prednisolone	20	TCMR Banff 1B	Methylprednisolone, ATG	Good graft function at 6 months
2	Deceased	1,2,2,1,1	ATG	MMF, tacrolimus, prednisolone	2	Borderline suspicious TCMR and suspicious ABMR	Methylprednisolone, IVIG	Good graft function at 20 months
3	Deceased	2,2,2,2,1	Alemtuzumab	MMF, tacrolimus, prednisolone	(a) 6 (b) 19	(a) TCMR and BK virus nephropathy (class 2) (b) TCMR and BK virus nephropathy (class 3)	(a) IVIG, his maintenance therapy is changed to sirolimus and leflunomide (b) methylprednisolone, sirolimus shifted to cyclosporine	Graft loss
4	Mother	0,0,0,0,0	Basiliximab	MMF, tacrolimus, prednisolone	36	Suspicious for ABMR, borderline suspicious for TCMR	Methylprednisolone, IVIG, plasma exchange, bortezomib	Good graft function at 19 months
5	Father	1,1,1,1,1,	Basiliximab	MMF, tacrolimus, prednisolone	1	TCMR (Banff 2A)	Methylprednisolone, IVIG, plasma exchange, alemtuzumab	Good graft function at 77 months
6	Deceased	1,2,2,2,1	Alemtuzumab	MMF, tacrolimus, prednisolone	5	TCMR	Methylprednisolone, and increased the dose of MMF	Good graft function post treatment, then lost follow‐up
7	Mother	_	_	MMF, tacrolimus, prednisolone	1	TCMR. Two years later, kidney biopsy showed BK virus nephropathy.	Methylprednisolone, IVIG and ATG	Good graft function at 13 years
8	Uncle	_	_	MMF, tacrolimus, prednisolone	62	Chronic rejection grade 2	Methylprednisolone, IVIG	Impaired graft function at 8 years
9	Deceased	2,2,1,2,2	ATG	MMF, tacrolimus, prednisolone	(a) 8 (b) 14 (c) 22	(a‐b) TCMR (Banff 2A) (c) BK virus nephropathy	Methylprednisolone	Impaired graft function at 36 months
10	Father	1,1,1,0,0	Alemtuzumab	MMF, tacrolimus, prednisolone	(a) 15 (b) 18	(a) TCMR (Banff 1B), suspicious for ABMR. (b) ABMR, borderline suspicious for TCMR.	(a) Methylprednisolone, IVIG, plasma exchange, ATG, rituximab, alemtuzumab. (b) IVIG, plasma exchange, rituximab, bortezomib.	Passed away 2 months later
11	Mother	1,1,1	Basiliximab	MMF, tacrolimus, prednisolone	(a) 86 & 92 (c) 96	(a & b) TCMR (Banff 1A) (c) Borderline suspicious TCMR and ABMR	(a &b) Methylprednisolone (c) Methylprednisolone, IVIG	Impaired graft function at 36 months
12	Mother	0,0,0,1,0	Basiliximab	MMF, tacrolimus, prednisolone	(a) 63 months (b) 64 months (c) 76 months	(a)TCMR (b) Suspicious for ABMR and borderline suspicious for TCMR (c) Active ABMR, borderline suspicious for TCMR	(a) Methylprednisolone (b) Methylprednisolone, IVIG, plasma exchange, bortezomib. (c) Methylprednisolone, IVIG, bortezomib	Good graft function at 55 months
13	Father	_	Basiliximab	MMF, tacrolimus, prednisolone	1	TCMR (Banff 2A)	Methylprednisolone, ATG	Impaired graft function at 72 months
14	Mother	0,0,0,0,0	Basiliximab	MMF, tacrolimus, prednisolone	(a) 24 (b) 26 (c) 29 (d) 36 (e) 93	(a) TCMR (Banff 1A) (b) ABMR (c) ABMR, Borderline TCMR (d) Borderline TCMR (e) TCMR (Banff 1B), ABMR	(a) Methylprednisolone. (b) Methylprednisolone, alemtuzumab. (c) IVIG, plasma exchange, bortezomib (d) Methylprednisolone. (d) Methylprednisolone, IVIG, ATG.	Impaired graft function at 16 months

Abbreviations: ABMR, antibody‐mediated rejection; ATG, antithymocyte globulin rabbit; IVIG, intravenous immunoglobulin; MMF, mycophenolate mofetil; TCMR, T cell–mediated rejection.

The 1‐ and 5‐year patient survival rates were 100% and 96.4%, respectively. Correspondingly, the graft survival rates were 98.1% and 89.2%. Mean eGFR at 1 and 5 years was comparable among induction groups (basiliximab, ATG, and alemtuzumab; 1‐year *p*‐value 0.51, 5‐year *p*‐value 0.46).

Overall, the prevalence of EBV, CMV, and BKV infection in the first year was 43%, 39%, and 33%, respectively, falling to 38%, 18%, and 12%, respectively, in the subsequent years. These rates did not differ significantly when stratified by induction regimen (basiliximab, ATG, and alemtuzumab; all *p*‐values > 0.25).

Thirty‐four (49%) children had at least one episode of culture‐positive urinary tract infection. Twelve children had UTIs within 6 months prior to their rejection episodes.

## 4. Discussion

The patient and graft survival at 1 and 5 years in our study have been consistent with published registry data [23]. The incidence of biopsy‐proven acute rejection was 23% in the first year, with a cumulative incidence of 36% at 5 years. These results are comparable to a recent report showing a rejection incidence of 23% at 1 year and 39% at 5 years [2, 10]. From 2012 to 2017, the North American Pediatric Renal Trials and Collaborative Studies (NAPRTCS) reported first‐year rejection rates of 12.7% from living donors and 13.2% from deceased donors, while the 2021 Scientific Registry of Transplant Recipients (SRTR) data showed varying rates by age, from 7.6% for ages 1–5, 10.9% for ages 6%–11%, and 13.0% for ages 12–17 [11, 12].

The presence of DSA is shown to be an independent predictor of graft rejection in this study. Its prevalence (28%) is similar to that previously reported (17%–39%), with the majority of the detected alloantibodies being Class II (DR and DQ) [13, 14, 20]. Also, as previously shown, this study confirms that the presence of two DR mismatches (in contrast to two DQ mismatches) predicts graft rejection (*p*‐value = 0.029) [15–17]. Two DR mismatches, therefore, should be avoided in donor selection for pediatric kidney transplants [10, 16].

It is worth noting that 20% of the graft rejections were DSA‐negative. As previously suggested, these cases could be attributed to non‐HLA alloantibodies, such as those against angiotensin II Type 1 receptor, endothelin‐1 Type A receptor, and endothelin [18, 19]. Thus, screening for non‐HLA alloantibodies may be necessary.

It is noteworthy that despite adopting the most up‐to‐date immunosuppression strategies and a shorter cold ischemia time over the course of the study period, the rate of rejections remains high. This may be attributable to a smaller population size and the wide heterogeneity of the deceased donor ethnicity, resulting in higher mean HLA mismatches, including HLA‐DR mismatches. Implementation of a national waiting list for children and a national HLA‐based allocation may help to improve the HLA matching and outcome [23].

The marked decline in eGFR in children with rejection, as shown in Figure 1, confirms acute rejection as a key determinant of long‐term kidney transplant outcomes, highlighting the need for enhanced surveillance and intervention to mitigate rejection’s adverse effects [21, 22].

## 5. Conclusions

Patient and graft survival at 1 year (100% and 98.1%) and at 5 years (96.4% and 89.2%) were comparable to the published registry data. Biallelic DR mismatch and the presence of DSA predicted the risk of graft rejection. Avoiding two DR mismatches, adherence to DSA surveillance, screening for non‐HLA antibodies, and optimizing immunosuppression can improve outcomes.

**Nomenclature**
SKMC: Sheikh Khalifa Medical City
ESRD: End‐stage renal disease
DSA: Donor‐specific antibody
EBV: Epstein–Barr virus
CMV: Cytomegalovirus
BKV: BK virus
ATG: Antithymocyte globulin
HLA: Human leukocyte antigen
IVIG: Intravenous immunoglobulin

## Ethics Statement

This study was approved by the SKMC Research Ethics Committee (857).

## Consent

Informed consent to participate in this retrospective chart review was exempted.

## Disclosure

All authors reviewed, edited, and agreed on the final submitted version of the manuscript. The abstract/data presented at the International Pediatric Summit on April 26, 2024.

## Conflicts of Interest

The authors declare no conflicts of interest.

## Author Contributions

All authors participated in the intellectual content, conception, and design of the paper and have agreed to have their names listed as contributors. Ela Beyyumi curated the data, performed the data analysis, and wrote the initial manuscript. Watfa Al Dhaheri suggested conducting the review. Muhammad Badar Zaman assisted in the literature review. Niaz Ahmad reviewed and edited the manuscript. Eihab Al Khasawneh conceptualized the study and suggested the initial review, emphasizing the main outcome data.

## Funding

No funding was used in this study.

## Data Availability

The data that support the findings of this study are available on request from the corresponding author. The data are not publicly available due to privacy or ethical restrictions.
